# Cortisol Rapidly Facilitates Glucocorticoid Receptor Translocation to the Plasma Membrane in Primary Trout Hepatocytes

**DOI:** 10.3390/biology12020311

**Published:** 2023-02-14

**Authors:** Chinmayee Das, Mathilakath M. Vijayan

**Affiliations:** Department of Biological Sciences, University of Calgary, Calgary, AB T2N 1N4, Canada

**Keywords:** rainbow trout, fish, GR, membrane fluidity, CRAC channel, intracellular calcium

## Abstract

**Simple Summary:**

Glucocorticoids elicit rapid cell signalling that is independent of the activation of the glucocorticoid receptor (GR) as a transcription factor. However, the mechanisms for this rapid nongenomic signalling by glucocorticoids are unclear. Here, we show that cortisol rapidly causes the translocation of intracellular GR to the plasma membrane in hepatocytes within minutes, and this response is mediated by the entry of extracellular calcium. We propose that this rapid cortisol action may sensitize the cells to nongenomic signalling via membrane-anchored GR.

**Abstract:**

Glucocorticoids (GCs) stimulate rapid cell signalling by activating the membrane-anchored intracellular glucocorticoid receptor (GR). However, the recruitment of the GR to the plasma membrane to facilitate nongenomic signalling is far from clear. As cytosolic free calcium ([Ca^2+^]i) is involved in intracellular protein dynamics, we tested the hypothesis that acute elevation in cortisol levels rapidly stimulates GR translocation to the plasma membrane via a calcium-dependent process in rainbow trout (*Oncorhynchus mykiss*) hepatocytes. To test this, we monitored temporal changes in intracellular GR distribution in response to cortisol exposure. Immunofluorescence labelling showed that the GR was present in cytosolic and nuclear compartments in trout hepatocytes. However, upon cortisol exposure, the GR rapidly (within 5 min) formed punctate and colocalized with caveolin-1, suggesting plasma membrane localization of the receptor. This redistribution of the GR to the plasma membrane was transient and lasted for 30 min and was evident even upon exposure to cortisol-BSA, a membrane-impermeable analogue of the steroid. The rapid cortisol-mediated GR translocation to the plasma membrane involved F-actin polymerization and was completely abolished in the presence of either EGTA or Cpd5J-4, a calcium release–activated calcium (CRAC) channel blocker. Additionally, the modulation of the biophysical properties of the plasma membrane by cholesterol or methyl β-cyclodextrin, which led to changes in ([Ca^2+^]i) levels, modified GR translocation to the plasma membrane. Altogether, acute cortisol-mediated rise in ([Ca^2+^]i) levels rapidly stimulated the translocation of intracellular GR to the plasma membrane, and we propose this as a mechanism promoting the nongenomic action of the GR for hepatocyte stress resistance.

## 1. Introduction

Stressors elevate circulating glucocorticoid (GC) levels, and this conserved stress response plays an important role in acute stress adaptation by facilitating energy substrate mobilization [1,2]. GC action is mediated by two intracellular receptors, the glucocorticoid receptor (GR) and the mineralocorticoid receptor (MR), both of which are ligand-bound transcription factors [3]. Most of the energy substrate mobilization role associated with stress coping has been ascribed to the activation of the GR by GCs [2]. In addition to its role in transcriptional regulation, the GR also plays a role in the rapid nongenomic signalling by GCs [4]. While the mechanism is unclear, the GC effect is thought to be mediated through a putative membrane GR (MbGR) [5]. However, to date, a membrane-GR-like protein has not been cloned or sequenced in any animal model [6,7]. 

One possibility for MbGR is the recruitment of intracellular GR to the plasma membrane by a yet unknown mechanism. For instance, receptors for the sex steroids, including estrogen receptor (ER), androgen receptor (AR), and progesterone receptor (PR), are translocated to the plasma membrane in mammalian models [8], and this involves the palmitoylation of the ligand binding domain of the receptor [8,9]. The recruitment of ER to the plasma membrane is associated with the lipid raft caveolar-rich membrane fractions [10,11]. Cav-1, a major caveolar marker protein, plays an important role in membrane-bound steroid-receptor-mediated nongenomic signalling [12,13], including the rapid activation of downstream protein kinases [14,15]. However, palmitoylation is not considered to play a role in GR recruitment to the plasma membrane [16].

We previously showed that cortisol rapidly activates protein kinases, including protein kinase C, in rainbow trout (*Oncorhynchus mykiss*) hepatocytes [17,18]. While these studies suggest rapid nongenomic signalling by cortisol, the membrane receptor(s) mediating these changes are unknown. A recent study in trout myocytes proposed a putative MbGR that might be involved in the rapid cortisol-mediated nongenomic responses [19]. As the cortisol-mediated rapid cellular responses were abolished with RU486 [19], a GR antagonist, this suggests the possibility that the intracellular GR anchored to the plasma membrane may be mediating the rapid response. This led us to the hypothesis that the intracellular GR may be rapidly recruited to the plasma membrane in response to elevated cortisol levels and may be a mechanism facilitating rapid nongenomic signalling. 

A key player modulating intracellular protein trafficking is the cytosolic free calcium ([Ca^2+^]i) levels. For instance, ([Ca^2+^]i) increase enabled the membrane localization of cytosolic phospholipase A2 in Chinese hamster ovary cell lines [20]. Additionally, a rise in ([Ca^2+^]i) mediated the translocation of cytosolic Ras-related C3 botulinum toxin substrate (Rac) to the plasma membrane in NIH 3T3 cells [21]. We recently showed that cortisol rapidly stimulated ([Ca^2+^]i) influx in trout hepatocytes, and this was mediated by the activation of calcium release–activated calcium (CRAC) channels [22]. The modulation of ([Ca^2+^]i) influx by CRAC channels is considered a conserved mechanism, especially in nonexcitable cells such as hepatocytes in mammals [23]. Ca^2+^ entry via CRAC is essential for many cellular responses, including intracellular protein dynamics [24], cytosolic 5-lipoxygenase enzyme recruitment to the nuclear membrane [25,26], and actin reorganization and dynamics [27]. However, whether Ca^2+^ entry via CRAC plays a role in steroid nuclear receptor trafficking is currently unknown. 

Given that cortisol rapidly increases ([Ca^2+^]i) in trout hepatocytes [22], we tested the hypothesis that this stress steroid triggers rapid GR redistribution to the plasma membrane in a calcium-dependent manner to facilitate rapid nongenomic signalling for stress resistance. We tested whether the membrane localization of the GR targets lipid rafts, and this was carried out by colocalizing membrane GR expressions with Cav-1, a caveolar marker protein of lipid-rich rafts [28]. Furthermore, the study assessed the role of membrane cholesterol content and F-actin cytoskeleton in regulating GR redistribution in trout hepatocytes. 

## 2. Materials and Methods

### 2.1. Reagents

Cortisol, EGTA (ethylene glycol bis (β-amino ethyl ether)-N,N,N′,N′-tetraacetic acid), trypan blue, cholesterol, methyl-beta-cyclodextrin (MβCD), latrunculin B, and Cpd5J-4 were purchased from Sigma, St. Louis, MO, USA. Pluoronic^®^-F 127 (20% solution in DMSO), Fura 2-AM, and Laurdan were purchased from Thermofisher, Waltham, MA, USA. Cortisol-conjugated BSA was purchased from EastCoast Bio, North Berwick, ME, USA. All other chemicals were of analytical grade and were purchased from local suppliers. 

### 2.2. Antibodies

The antibodies used in the present study were rabbit polyclonal antibody against rainbow trout GR (1:250) [29], Caveolin-1 mouse monoclonal antibody (1:500) [30,31], ORAI-1 rabbit polyclonal antibody (1:250) [22,32,33], and phalloidin conjugated TRITC (1:500) for F-actin detection (1:500) [34]. The secondary antibodies used were Alexa 488 goat anti-rabbit IgG and Alexa 594 donkey anti-mouse IgG (Invitrogen, Burlington, ON, Canada).

### 2.3. Animals

Juvenile rainbow trout (~200–300 g body mass) species were obtained from the Allison Creek, Brood Trout Station, Alberta Environment and Parks (Crowsnest Pass, AB, Canada). The fish were maintained at the Biological Sciences Animal Facility, University of Calgary, for at least two weeks prior to the isolation of hepatocytes. They were held in a 500 L tank supplied with a constant flow of aerated de-chlorinated water (12 ± 2 °C), with the photoperiod set at 12 h light:12 h dark. The fish were fed commercial trout feed (Martin Mills, Elmira, ON, Canada) to satiety once daily, and they were food-deprived for 24 h prior to hepatocyte isolation. A total of 10 fish (biological replicates) were used for the hepatocyte isolation study. The experimental procedures were approved by the animal care committee at the University of Calgary and were in accordance with the Canadian Council on Animal Care guidelines. 

### 2.4. Hepatocyte Isolation and Cell Suspension

Hepatocytes were isolated following the collagenase digestion procedure described previously [35]. In brief, fish were anaesthetized using 2-phenoxyethanol (Thermofisher) at a concentration of 600 µL/L followed by cannulation of the hepatic portal vein, as described previously [22]. The liver was then perfused with Medium A (in mM), NaCl 136.9, KCL 5.4, MgSO_4_·7H_2_O 0.8, Na_2_HPO_4_·12H_2_O 0.33, KH_2_PO_4_ 0.44, HEPES 5.0, and HEPES Na 5.0) to remove blood followed by Medium A with collagenase (Medium B: Medium A plus 5 mg/10 mL collagenase; Thermofisher) for 20–30 min. The buffers used for hepatocyte isolation were kept on ice throughout the process. After perfusion, the liver was finely minced using a sterile scalpel and transferred to a Petri dish with Medium A. The suspension was filtered through two nylon meshes (250 and 75 µm, respectively) and the cell suspension was centrifuged (MultifugeX3R centrifuge, Thermofisher) at 200 g for 5 min at 11–12 °C, and this was repeated three times to wash the cells of residual collagenase. The pellet was resuspended in Medium A containing calcium and BSA (Medium C: Medium A with 1.5 mM CaCl_2_ and 1% BSA) and centrifuged as mentioned above. The final pellet was resuspended in 25 mL of L-15 medium (Leibovitz’s medium; Gibco, Thermofisher) with 5 mM NaHCO_3_ and antibiotic/antimycotic supplementation and was allowed to settle on ice for 30 min. The cell pellet was then resuspended in 10 mL of L-15 medium followed by cell counting using a haemocytometer. Cell viability was checked using a trypan blue (Sigma) dye exclusion test, and the viability was always >95%. 

### 2.5. Immunofluorescent Labelling

The isolated hepatocytes were plated on 22 mm coverslips in 6-well Petri plates at a concentration of 0.75 × 10^5^ million cells per ml in L-15 medium at 11 °C for 24 h prior to the treatments. Hepatocytes were then exposed to cortisol at a concentration of 100 ng/mL in a time-dependent (5, 10, 30, or 60 min) manner, following which they were fixed in ice-cold methanol for immunostaining. All pharmacological manipulation, including cholesterol (1 mM) and MβCD (10 mM), EGTA (2 mM), and Cpd5J-4 (10 µM), were carried out in a serum-free medium at 11 °C prior to cortisol addition. The coverslips were then fixed using ice-cold methanol and stored at 4 °C. Excess methanol was removed, and a hydrophobic pen was used to draw a barrier around the coverslip boundary to secure the central area for antibody application. The coverslips with cells attached were then incubated with a permeabilization buffer (0.1% TritonX-100 in Medium A) for 10 min followed by washing with a buffer solution (Medium A with 0.1% Tween-20) 3 times at 5 min intervals. After permeabilization, the cells were incubated with a blocking solution (2% BSA with sodium azide in Medium A) for 3 h at 37 °C followed by overnight incubation with anti-trout GR antibody [29] at 4 °C. After overnight incubation, the cells were washed as previously mentioned followed by secondary antibody goat anti-rabbit Alexa 488 conjugate labelling [36]. Double-antibody staining was followed by incubating the cells with anti-Cav-1 antibody (1:250) [30,31] overnight at 4 °C. Secondary antibody donkey anti-mouse Alexa 594 conjugate labelling [37] was performed the very next day after washing. DAPI (100 ng/mL) is the final staining performed prior to mounting. The coverslips were mounted to clean the slides using DABCO (Antifade reagent) followed by sealing the periphery using transparent nail paint. This whole mounting step lasted for an hour including air-drying. Post-drying, the slides were wrapped in aluminium foil and stored at 4 °C until imaging. This step was crucial for keeping the staining process stable for a longer period with less photobleaching.

**Figure 1:**
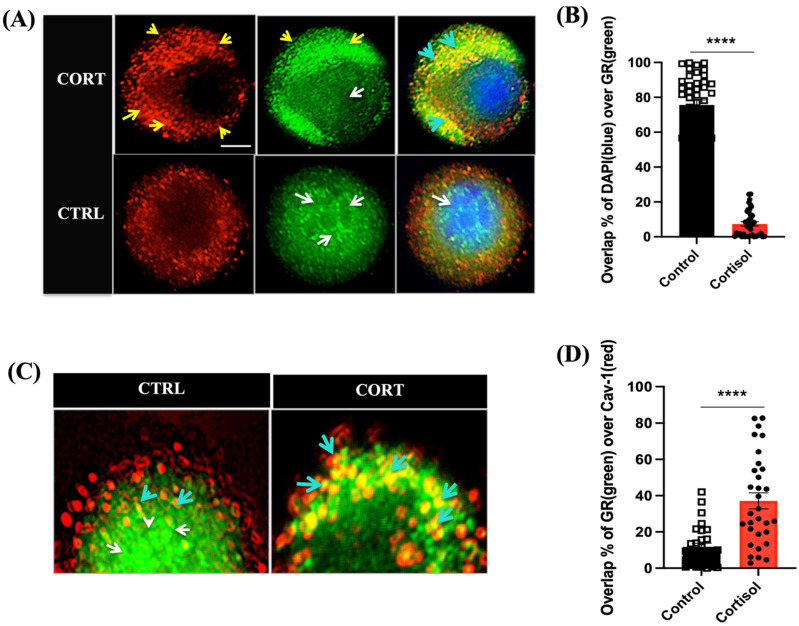
Cortisol rapidly translocates GR to the plasma membrane in trout hepatocytes: (**A**) within 5 min after cortisol exposure, GR (green) rapidly moves away from the nucleus and forms punctate at the plasma membrane (The top panel); (**B**) the fluorescence intensity overlaps between the nuclear (DAPI) and GR signal (green) were quantified (see Methods) and are shown as percentage colocalization, which is suggestive of nuclear localization in the control and cortisol-exposed cells. (**C**) Colocalization of GR with Cav-1 (red) was obtained using colocalization threshold/coloc-2 plugin platform of ImageJ and is seen as yellow. Control cell on the left showed very little colocalization with caveolin-1 compared with the cortisol-treated cell on the right (the cyan arrows indicate colocalization). (**D**) The fluorescence intensity overlaps between the GR and Cav-1 signal were quantified (see Methods) and are shown as percentage overlap, which is suggestive of plasma membrane localization, in the control and cortisol-exposed cells. GR (green), caveolin-1 (red) and DAPI (blue); yellow arrows indicate GR localization at the membrane; white arrows indicate nuclear localization; cyan arrows denote colocalization of GR with Cav-1; scale bar: 25 μm. Abbreviations: control (CTRL), cortisol (CORT), caveolin-1(Cav-1), glucocorticoid receptor (GR). **** *p* < 0.0001 (*n* = 50 individual cells from 5 different fish; Student’s *t*-test).

The spatial colocalization of fluorescently labelled proteins of interest was carried out using JACOP(FIJI). Due to the inbuilt ability of image thresholding and background subtraction in the JACOP plugin, it worked as an appropriate tool for our study. Following thresholding, Mander’s correlation coefficient was used to determine the intensity of overlap between two channels at a particular region of interest (ROI) with background subtraction [38]. The further overlap intensities that were determined were averaged across the experiments. The superimposition of channels helped in determining the spatial distribution of the GR with Cav-1 (Figure 1), and colocalization percentage analysis using Mander’s coefficient [38] indicated the proximity shared between the two protein binding regions and the degree of spatial coherence in the cortisol group (Figure 1).

### 2.6. Cytosolic Ca^2+^ Measurements

The cytosolic Ca^2+^ measurements were carried out as described previously [22]. Briefly, hepatocytes were incubated with FURA-2 AM (10 µM in Medium C) and 5 µL of 20% pluronic-F127 in DMSO for 1.5 h followed by washing and centrifugation at 200 g for 5 min. Cell viability was analyzed using a trypan blue dye exclusion test before and after each treatment. The cell pellet was resuspended in Medium C for 30 min for the cell esterases to carry out de-esterification followed by imaging. For treatments, the cells were pre-incubated with cholesterol (1 mM) and MβCD (10 mM) for 1 h in a serum-free medium and then exposed to cortisol (100 ng/mL) prior to cell Ca^2+^ recording. Cell suspension (50 µL) was mounted on a coverslip (of approx. 18 mm) attached to a Petri dish of 28 mm diameter constructed in the lab [39]. This whole setup was then mounted on an inverted microscope (Qimaging RETIGA^TM^ EXi FAST 1394, Ontario, NY, USA) for imaging. The cells were imaged for 10 min at room temperature (~20 °C) for Ca^2+^ recording with an interval of 10 s in between each recording. Intracellular calcium was expressed as a ratio of fluorescence intensities at 380 nm (free calcium) and 340 nm (bound to calcium), as described previously [22]. Image acquisition was computer-controlled by Northern Eclipse (EMPIX, Imaging, Mississauga, ON, Canada), and images were acquired at 10 s intervals to reduce photobleaching. 

### 2.7. Laurdan Generalized Polarization Imaging and Quantification

Cells were pre-incubated with cholesterol (1 mM) and MβCD (10 mM) for 1 h at 11 °C in a serum-free medium before Laurdan (10 µM) [40] probing. All the incubation steps were carried out in a black 96-well plate (Greiner Bio-One, Monroe, NC, USA). The cells pre-treated with cholesterol and MβCD were used for basal reading before cortisol addition. Plate readings were taken at 19~20 °C for 10 min with 30 s intervals between each reading. Data analysis was carried out by subtracting the background recordings from control cells with a Laurdan probe. Data represent reading at 10 min post-cortisol exposure.

Laurdan is a membrane probe, which is used widely to understand the membrane microarchitecture by relating the percentage of water retention in the membrane, which in turn affects the probe movement and signal. Changes to the dipole moment define the intramembranous environment. Thus, a slight change in the membrane microarchitecture not only will affect the rotation of Laurdan but also the generalized polarization (GP) process. Laurdan anisotropy measurements were taken using a spectro-fluorimeter (Spectra-max Paradigm; Molecular Devices, San Jose, CA, USA). The prepared sample plates were observed at 350 nm excitation wavelength and two different emission wavelengths 440 and 490 nm, respectively. Changes to the membrane parameters were calculated using generalized polarization (GP) shift. The values obtained under different emission intensities were calculated using the following equation adapted from Parassasi et al. (1991) [41]:

GP = I_440_ − I_490_/I_440_ + I_490_ (1)

### 2.8. Data Acquisition and Analysis

The ion-wave acquisition tool of Northern Eclipse software was used for ratiometric calcium imaging. The region of interest (ROI) was selected for each experiment with the respective background area. The data obtained using the ion-wave acquisition tool of Northern Eclipse software were plotted on SIGMA plot against time (in seconds) to obtain the time-lapse changes in calcium and changes seen within a minute of cortisol stimulation, as described previously [22]. The images were created using ImageJ with a LUT of 16 colors for intensity changes. Statistical significance was determined using Student’s *t*-test or one-way ANOVA followed by a post hoc test (Tukey’s multiple-comparison test) to determine treatment differences. The equal variance was tested using Levene’s median test, and normality was tested using the Shapiro–Wilk test. *p* < 0.05 was considered significant.

## 3. Results

### 3.1. Cortisol Rapidly Translocate GR to the Plasma Membrane in Trout Hepatocytes

Immunolabelling with a trout-specific GR antibody was used to determine the spatial and temporal distribution of GR post-cortisol stimulation. The GR was localized in the cytoplasm and nucleus of trout hepatocytes (control group, Figure 1A) prior to cortisol addition. Within 5 min of cortisol exposure (100 ng/mL), a concentration that is measured in the plasma of acutely stressed rainbow trout [18], the GR rapidly formed punctate at the plasma membrane (Figure 1A). Colocalization analysis using Mander’s coefficient [38] showed a significantly (*p* < 0.0001) lower overlap between DAPI (blue), a nuclear signal, and the GR (green) in the cortisol-treated cells compared with the control cells (Figure 1B). To further confirm whether the GR redistribution was at the plasma membrane, double immunolabelling was carried out with the GR and caveolar membrane marker protein, Cav-1, which is abundant in the caveolae-rich areas of the plasma membrane [28]. After 5 min of cortisol exposure, there was a higher membrane localization of the GR with Cav-1 (the yellow color: cyan arrows) than the control cells, which showed a predominantly nuclear and cytosolic distribution of the GR (green) and not around the membrane (Figure 1C). Colocalization analysis using Mander’s coefficient [38] showed a significantly (*p* < 0.0001) higher overlap between the GR (green) and Cav-1 (red) in the cortisol-exposed cells compared with the control cells (Figure 1D). 

### 3.2. Cortisol Mediates Temporal Changes in GR Distribution in Trout Hepatocytes

**Figure 2:**
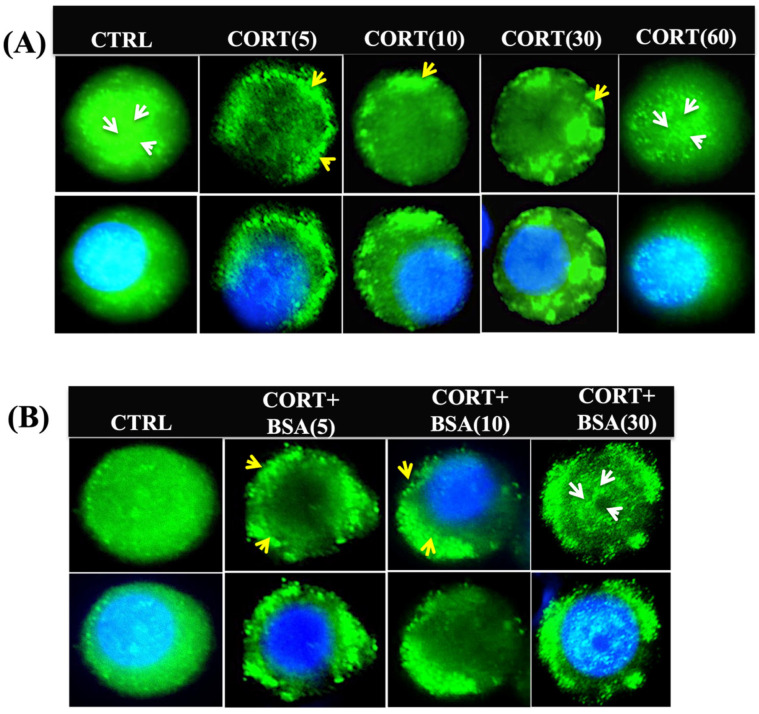
Cortisol mediates temporal changes in GR distribution in trout hepatocytes: (**A**) representative images showing temporal changes (5, 10, 30, and 60 min) in GR expression post-cortisol (100 ng/mL) stimulation. Trout GR was detected with a specific anti-trout GR antibody at a dilution of 1:250. The upper panel demonstrates GR expression, and the lower panel indicates merged images of GR with the nuclear stain DAPI; (**B**) temporal changes (5, 10, and 30 min) in GR localization in the presence of the membrane-impermeable steroid analogue (cortisol-BSA). GR (green) and DAPI (blue); yellow arrows indicate GR localization and punctae formation at the membrane, while white arrows indicate nuclear localization. Sample size: 100 individual cells from 5–6 different fish. Scale bar: 25 μm. Abbreviations: control (CTRL), cortisol (CORT), cortisol-conjugated BSA (CORT + BSA), and the time in min is shown in parentheses.

The GR recruitment to the plasma membrane was transient (Figure 2A), showing a rapid increase at 5 min, which was maintained for 30 min, followed by a return to control distribution at 60 min post-cortisol exposure (Figure 2A). To determine whether the cortisol-induced rapid GR redistribution was mediated by membrane signalling, a membrane-impermeable ligand (cortisol-BSA) was also utilized. Exposure to cortisol-BSA (100 ng/mL) also led to GR localization on the plasma membrane. The GR rapidly formed the punctate at the membrane by 5 min of cortisol-BSA addition, and this response was still evident 30 min post-treatment (Figure 2B). 

### 3.3. Cortisol-Mediated Membrane’s GR Localization Is Calcium-Dependent

**Figure 3:**
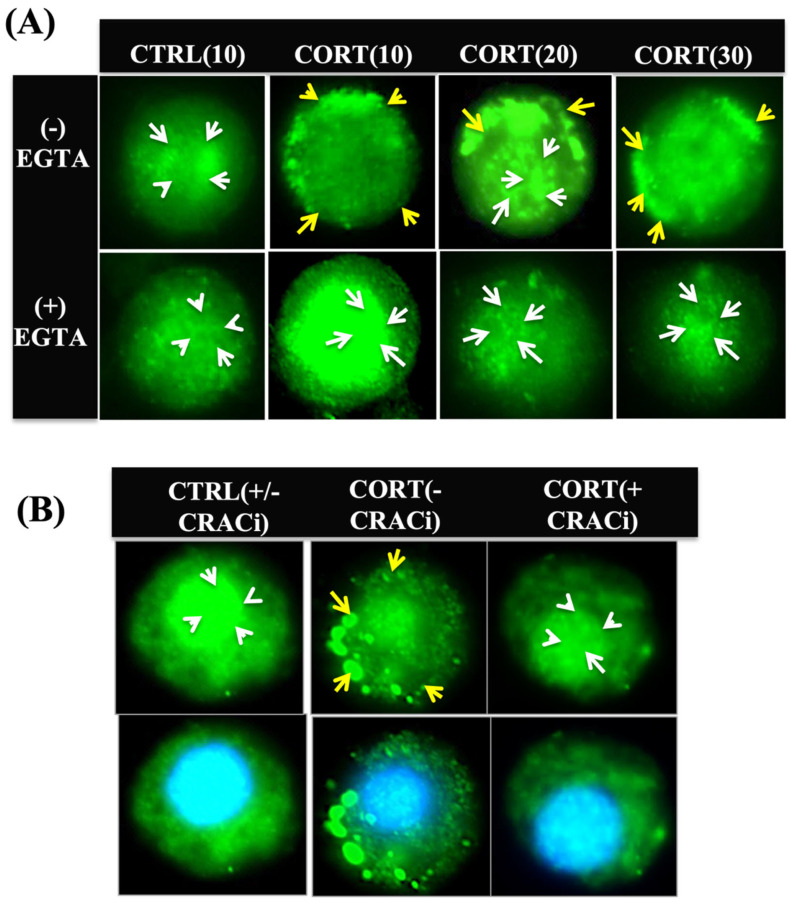
Cortisol-mediated membrane GR localization is calcium-dependent: (**A**) extracellular calcium chelation with EGTA inhibited the temporal (10, 20, and 30 min) GR localization at the plasma membrane seen with cortisol; representative images without EGTA is shown on the upper panel, while cells with EGTA are on the lower panel; (**B**) GR recruitment to the plasma membrane with cortisol at 5 min is also absent in the presence of the ORAI-1 inhibitor, Cpd5J-4; upper panel shows control and cortisol group either with or without Cpd5J-4, while the lower panel shows the merged image with the DAPI stained nucleus; GR (green) and DAPI (blue); yellow arrows indicate GR localization at the membrane, while white arrows indicate nuclear localization. Sample size: 50 individual cells from 5 different fish. Scale bar: 25 μm. Abbreviations: control (CTRL), cortisol (CORT), ethylene glycol-bis(β-aminoethyl ether)-N,N,N′,N′-tetraacetic acid (EGTA), Cpd5J-4 (Calcium release–activated calcium channel inhibitor; CRACi).

To determine whether an increase in ([Ca^2+^]i) due to cortisol stimulation was involved in this GR recruitment to the plasma membrane, we manipulated ([Ca^2+^]i) levels by using EGTA (an extracellular calcium chelator) or blocking the ORAI-1, the CRAC channel protein subunit [22,42]. The treatment of hepatocytes with EGTA (2 mM) for 30 min, which prevents the rapid rise in ([Ca^2+^]i) with cortisol [22], abolished the redistribution of the GR to the plasma membrane (Figure 3A). Additionally, the hepatocytes pre-incubated with the CRAC channel blocker, Cpd5J-4 (10 μM), for 30 min, which prevented the rapid rise in ([Ca^2+^]i) with cortisol [22], also abolished the GR punctate formation at the plasma membrane (Figure 3B). 

### 3.4. Cortisol-Mediated GR Translocation Involves F-Actin Framework

**Figure 4:**
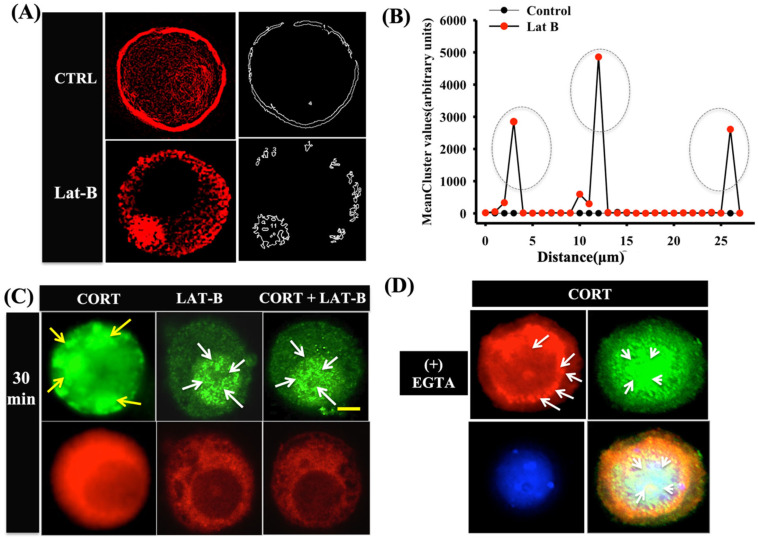
Cortisol-mediated GR translocation involves F-actin framework: (**A**) representative image showing a deconvoluted intact control cell (upper panel) and a latrunculin-B-treated cell (lower panel); immunofluorescence staining with phalloidin TRITC (red) denote condensed structures and actin clustering in the latrunculin B treatment (Left column); (**B**) mean cluster plot indicating the cluster sizes formed between control and latrunculin-B-treated cells (see Methods). Peaks represent clusters and are indicated by circles; (**C**) representative images showing GR translocation post-cortisol stimulation in the control and latrunculin-B-treated cells. The upper panel denotes changes to GR expression at 30 min post-cortisol treatment either with or without latrunculin-B treatment. The lower panel shows F-actin (phalloidin TRITC) staining; (**D**) representative cell images demonstrating the effect of EGTA on F-actin (using phalloidin) staining; GR (green), F-actin (red), and DAPI (blue); yellow arrows indicate GR localization at the membrane, while white arrows indicate nuclear localization. Sample size: 100 individual cells from 5 different fish. Scale bar: 25 μm. Abbreviations: control (CTRL), cortisol (CORT), ethylene glycol-bis(β-aminoethyl ether)-N,N,N′,N′-tetraacetic acid (EGTA), latrunculin B (LAT-B).

To determine whether cortisol-mediated GR translocation requires the involvement of cytoskeletal filaments, we treated the hepatocytes with latrunculin B (F-actin disruptor). The hepatocytes were pre-incubated with latrunculin B [43] for 40 min prior to cortisol stimulation. Due to the distinct condensed actin structure in the hepatocytes, it was difficult to detect dynamic changes to actin reorganization. Hence, confocal imaging and deconvolution tool was used to determine the sharp structural differences between the treatment groups prior to and post-exposure to latrunculin B. Rhodamine-labelled phalloidin stain was used to detect the changes to actin organization at a wavelength of 594 nm [44]. The addition of latrunculin B resulted in the formation of actin clusters inside the hepatocytes (Figure 4A). This was further confirmed by using cell surface clustering via the Image J3D object counter tool, which showed three distinct clustering peaks with latrunculin but not in the controls (Figure 4B). Latrunculin B pre-treatment completely failed to translocate the GR to the membrane post-cortisol stimulation (Figure 4C). Increased GR expression was seen in the nucleus relative to the cytoplasm in the latrunculin-B-treated cells (Figure 4C). Chelating extracellular Ca^2+^ using EGTA (2 mM) for 30 min showed actin disassembly and failed to translocate the GR to the membrane (Figure 4D). 

### 3.5. Cortisol-Mediated GR Translocation Is Modulated by Plasma Membrane Modifications

**Figure 5:**
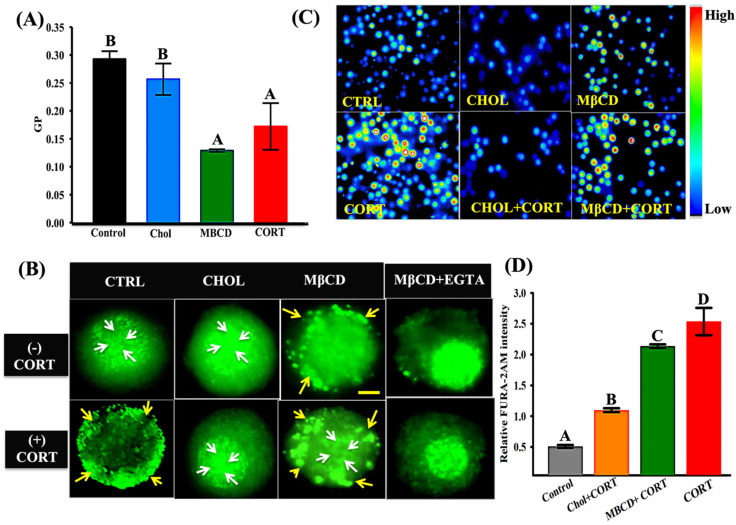
Cortisol-mediated GR translocation is modulated by membrane modifications: (**A**) Laurdan generalized polarization (GP) values for hepatocytes treated with either vehicle (control), cholesterol (CHOL), MβCD, or cortisol (CORT); (**B**) representative images of GR (green) localization in hepatocytes treated either with vehicle (CTRL), cholesterol (CHOL), or methyl-beta-cyclodextrin (MβCD) in the presence (lower panel) or absence (upper panel) of cortisol (CORT). The last panel shows GR localization in the MβCD group with EGTA either in the presence or absence of cortisol; (**C**) representative images of hepatocytes treated with either vehicle (CTRL), cholesterol (CHOL), or methyl-beta-cyclodextrin (MβCD) in the presence (lower panel) or absence (upper panel) of cortisol on iCa^2+^ levels using ratiometric imaging with FURA-2AM; (**D**) mean calcium fluorescence measured at 1 min after cortisol (CORT) addition in cells treated with vehicle (control), cholesterol (Chol), or methyl-beta-cyclodextrin (MβCD). Yellow arrows indicate peripheral GR localization; white arrows indicate nuclear localization. Bar represents mean ± SEM; different letters denote significant difference; one-way ANOVA (*n* = cells from 4–5 fish; *p* < 0.001). Scale bar: 25 μm. Abbreviations: control (CTRL), cortisol (CORT), methyl-beta-cyclodextrin (MβCD), cholesterol (CHOL), ethylene glycol-bis(β-aminoethyl ether)-N,N,N′,N′-tetraacetic acid (EGTA), generalized polarization (GP).

To determine whether plasma membrane modifications, including alteration in membrane fluidity, may impact GR localization, we manipulated the cells through pre-treatment with either MβCD and/or cholesterol [45]. To confirm the changes in membrane biophysical properties, Laurdan emission spectral alteration was measured. This was carried out by calculating the GP value, a measure of solvent relaxation and lipid disorder [46], from spectral changes at 440 and 490 nm. Cholesterol enrichment did not affect the GP compared with the control cells, while both MβCD and cortisol treatment significantly reduced the GP values compared with the control cells (Figure 5A). Cholesterol enrichment showed GR localization similar to that seen in the control cells (Figure 5B). Cholesterol depletion with MβCD resulted in the formation of GR punctae at the plasma membrane of trout hepatocytes similar to that seen with cortisol treatment (Figure 5B). This MβCD effect on GR localization was not altered by cortisol treatment (Figure 5B). To determine if Ca^2+^ influx was important for GR translocation in the MβCD-treated hepatocytes, extracellular Ca^2+^ was chelated with EGTA. In the absence of external Ca^2+^, the GR failed to translocate to the plasma membrane in the MβCD-treated cells (Figure 5B). There was a higher basal ([Ca^2+^]i) in the MβCD-treated cells, but not in the cholesterol-treated cells, compared with the control cells (Figure 5C). As shown before [22], acute cortisol exposure rapidly increases intracellular Ca^2+^ concentration in trout hepatocytes (Figure 5C,D). Cortisol exposure significantly increased ([Ca^2+^]i) in the MβCD-treated cells compared with the cholesterol-treated cells (Figure 5D). 

## 4. Discussion

This study demonstrates that the acute stress levels of cortisol promote the rapid translocation of intracellular GR to the plasma membrane in trout hepatocytes. Plasma-membrane-localized GR is thought to play a role in the rapid nongenomic signalling by GCs [4,16], including trout myocytes [19]. This may be mediated by the interaction of plasma membrane GR with Cav-1, a major scaffolding protein abundant in lipid-rich rafts, which facilitates nongenomic cell signalling [12,13,47]. For instance, the sex-steroid receptor translocates to the plasma membrane, interacts with Cav-1, and activates rapid signalling, and this process is thought to require palmitoylation within the ligand-binding domain in mammalian cells [8,15]. However, it appears that palmitoylation may not be the case for GR distribution to the plasma membrane [16]. Here, we show that the acute elevation of cortisol stimulates ([Ca^2+^]i) rise, either by modulating the CRAC channel activity [22] and/or by altering the biophysical property of the membrane (Figure 5), leading to the rapid redistribution of the GR to the plasma membrane in trout hepatocytes. 

The GR is a nuclear receptor family of proteins that are mostly localized to the cytoplasm/nucleus [15], and upon ligand binding, they initiate the transcriptional regulation of target genes [2,48,49,50]. This is also the case in trout hepatocytes in the present study, as the intracellular GR distribution was evident in both the cytosol and the nucleus. However, this pattern rapidly changed in response to cortisol stimulation, as the GR not only formed the punctae but was also localized to the plasma membrane. The intracellular redistribution of the GR in response to cortisol stimulation was rapid and transient and returned to pre-treatment distribution by 60 min post-cortisol treatment. The rapid formation of punctae and colocalization with Cav-1 at 5 min post-cortisol exposure correlated with a reduced overlap of the GR and DAPI in the nucleus, suggesting a redistribution of the nuclear GR (Figure 1B,D). The colocalization of the GR with Cav-1 (Figure 1C) suggests that the punctae formation was at the plasma membrane and localized to lipid rafts, which facilitates the rapid nongenomic signalling of GCs in mammalian cells [16,51]. In agreement, studies have shown that the rapid nongenomic actions of GCs are abolished by RU486, an intracellular GR antagonist, supporting a physiological role for the membrane anchoring of intracellular GR, including in fish myocytes [16,19]. Consequently, the rapid translocation of the GR to the plasma membrane and their association with lipid rafts may be a possible mechanism activating the rapid nongenomic actions of GCs during stress [19,52,53,54]. The fact that the GR redistribution to the plasma membrane was also evident with cortisol-BSA, which is a membrane-impermeable analogue of the steroid, further reinforces a membrane-mediated cortisol signalling in trout hepatocytes. 

We recently showed that cortisol rapidly elevates ([Ca^2+^]i) within minutes, and this is mediated by CRAC channel gating in trout hepatocytes [22]. Consequently, we tested whether Ca^2+^ may be involved in the rapid GR translocation to the plasma membrane by cortisol. Indeed, the chelation of extracellular Ca^2+^ prevented the redistribution of intracellular GR in trout hepatocytes. As CRAC channels are involved in the calcium influx from extracellular space in hepatocytes, blocking the channel with Cpd5J-4 [43] also abolished GR translocation to the plasma membrane (Figure 3B). These results support a key role for the cortisol-stimulated rapid increase in ([Ca^2+^]i) in the redistribution of the GR to the plasma membrane. Although intracellular Ca^2+^ increase has been shown to facilitate protein trafficking in mammalian cells [20,55], to our knowledge, this study is the first to attribute a role for ([Ca^2+^]i) rise in GR localization to the plasma membrane. 

Although Ca^2+^ is a key second messenger, the transient movement of protein between intracellular compartments also requires a cytoskeletal framework [56], including the translocation of protein from the intracellular compartment to the plasma membrane [57,58,59]. For instance, the translocation of ligand-bound GR to the nuclear compartment requires F-actin, and this nucleocytoplasmic shuttling failed after actin disruption in mammals [60]. Our results suggest that F-actin is also essential for the movement of cytosolic GR to the plasma membrane in trout hepatocytes (Figure 4), as the disruption of the F-actin network using latrunculin B prevented GR redistribution and punctae formation at the plasma membrane. This F-actin-mediated redistribution of the GR to the hepatocyte membrane may be a Ca^2+^-dependent process, as preventing extracellular Ca^2+^ entry abolished the cortisol-mediated GR localization to the plasma membrane and led to the clumping of F-actin (Figure 4). In agreement, studies have shown that the interaction of Ca^2+^ with the cytoskeleton, including tubulin [61] and actin filaments [62], plays a role in the translocation of proteins between intracellular compartments.

The plasma membrane’s biophysical properties also play roles in protein trafficking, sorting, and signal transduction across intracellular compartments [63,64]. For instance, cholesterol packaging determines the biophysical properties of the cellular membrane, maintains structural integrity, and facilitates cellular function, including membrane fusion, random protein distribution, and changes to the channel activity in mammalian cells [65,66]. We have previously shown that cortisol rapidly alters the membrane fluidity, leading to an increase in the phosphorylation of PKA, PKC, and AKT substrates in trout hepatocytes [17]. Here, we showed that alterations in the membrane properties affect GR redistribution to the plasma membrane, and this may be due in part to the modulation of ([Ca^2+^]i) levels. Increasing membrane fluidity by reducing cholesterol packing by MβCD led to the formation of GR punctae on the plasma membrane of trout hepatocytes. This response mimics cortisol stimulation and may be due to the higher basal ([Ca^2+^]i) levels in the MβCD-treated cells (Figure 5C). Cholesterol addition to the cholesterol-depleted cells prevented the cortisol-mediated GR translocation, and this corresponded to a lack of change in ([Ca^2+^]i) (Figure 5C). Indeed, cholesterol enrichment has been shown to reduce CRAC channel activity by reducing the channel selectivity at the TM1pore [67]. Given that cortisol increases membrane fluidity in trout hepatocytes [17], this may also play a role in CRAC channel gating and the rapid ([Ca^2+^]i) rise reported previously with cortisol [22]. Consequently, the mode of action of cortisol in the rapid redistribution of intracellular GR involves the modulation of ([Ca^2+^]i) levels in trout hepatocytes, and this is mediated either by alterations in the membrane biophysical properties (this study) and/or the direct regulation of CRAC channel activity [22]. 

## 5. Conclusions

**Figure 6:**
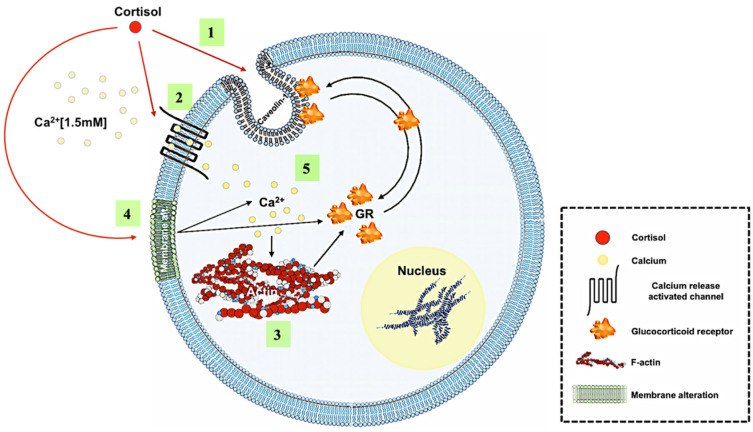
Proposed mechanism for cortisol-mediated rapid GR translocation to the plasma membrane: (1) cortisol rapidly localizes GR to the plasma membrane, and the GR colocalizes with caveolin-1; (2) rapid GR translocation is dependent on extracellular Ca^2+^ and involves ORAI-1 activation; (3) cortisol-mediated rapid GR translocation involves actin cytoskeletal network; (4) membrane modifications can alter GR translocation; (5) this may mediate changes in calcium dynamics in trout hepatocytes.

Taken together, our results demonstrate for the first time that cortisol rapidly redistributes the GR to the plasma membrane in trout hepatocytes. This is supported by the colocalization of the GR with Cav-1, a key membrane protein that is involved in transducing membrane GR-mediated rapid nongenomic signalling [68]. The results reveal that cortisol-mediated rapid rise in ([Ca^2+^]i) may be a key mechanism promoting the rapid GR redistribution to the plasma membrane (Figure 6). This ([Ca^2+^]i) rise may be due to the action of cortisol by either directly modulating CRAC channel activity [22] or indirectly by altering the fluidity of the plasma membrane. The ([Ca^2+^]i) rise may also facilitate F-actin polymerization, allowing for the redistribution of the GR in trout hepatocytes (Figure 6).

Overall, rapid GR redistribution to the plasma membrane by cortisol is a novel phenomenon and may enable rapid nongenomic signalling by membrane-anchored intracellular GR for stress coping in hepatocytes. Further studies are clearly warranted to understand the physiological significance of this rapid GR translocation and to investigate if this has a role in priming cells for stress adaptation.

## Data Availability

Data will be made available upon reasonable request.
